# A dataset comprising 141 magnetic resonance imaging scans of 98 extant sea urchin species

**DOI:** 10.1186/2047-217X-3-21

**Published:** 2014-10-14

**Authors:** Alexander Ziegler, Cornelius Faber, Susanne Mueller, Nina Nagelmann, Leif Schröder

**Affiliations:** 1 Ziegler Biosolutions, Fahrgasse 5, 79761 Waldshut-Tiengen, Germany; 2 Institut für Klinische Radiologie, Universitätsklinikum Münster, Albert-Schweitzer-Campus 1, 48149 Münster, Germany; 3 Centrum für Schlaganfallforschung, Charité-Universitätsmedizin Berlin, Charitéplatz 1, 10117 Berlin, Germany; 4 Molecular Imaging Group, Leibniz-Institut für Molekulare Pharmakologie, Robert-Rössle-Straße 10, 13125 Berlin, Germany

**Keywords:** MRI, Echinodermata, Echinoidea, Morphology, Soft tissue, Systematics, Anatomy, Repository, Evolution, Morphomics

## Abstract

**Background:**

Apart from its application in human diagnostics, magnetic resonance imaging (MRI) can also be used to study the internal anatomy of zoological specimens. As a non-invasive imaging technique, MRI has several advantages, such as rapid data acquisition, output of true three-dimensional imagery, and provision of digital data right from the onset of a study. Of particular importance for comparative zoological studies is the capacity of MRI to conduct high-throughput analyses of multiple specimens. In this study, MRI was applied to systematically document the internal anatomy of 98 representative species of sea urchins (Echinodermata: Echinoidea).

**Findings:**

The dataset includes raw and derived image data from 141 MRI scans. Most of the whole sea urchin specimens analyzed were obtained from museum collections. The attained scan resolutions permit differentiation of various internal organs, including the digestive tract, reproductive system, coelomic compartments, and lantern musculature. All data deposited in the *Giga*DB repository can be accessed using open source software. Potential uses of the dataset include interactive exploration of sea urchin anatomy, morphometric and volumetric analyses of internal organs observed in their natural context, as well as correlation of hard and soft tissue structures.

**Conclusions:**

The dataset covers a broad taxonomical and morphological spectrum of the Echinoidea, focusing on ‘regular’ sea urchin taxa. The deposited files significantly expand the amount of morphological data on echinoids that are electronically available. The approach chosen here can be extended to various other vertebrate and invertebrate taxa. We argue that publicly available digital anatomical and morphological data gathered during experiments involving non-invasive imaging techniques constitute one of the prerequisites for future large-scale genotype—phenotype correlations.

## Data description

### Purpose of data acquisition

Despite the fact that sea urchins (Echinodermata: Echinoidea) have served as model organisms in various biological disciplines for over a century and a half [1], the internal anatomy of this taxon had never been systematically analyzed on a large scale. Until recently, such broad inferences would invariably have required the undesirable dissection of valuable material, including museum type specimens. However, non-invasive scanning techniques such as magnetic resonance imaging (MRI) now permit studying echinoid internal anatomy without the need for dissection [2]. In addition to its suitability for studies on sea urchins, MRI can also be used to visualize the soft tissue anatomy of other invertebrate as well as vertebrate taxa [3]. Most importantly, MRI experiments result in digital data suitable for rapid online dissemination [4].

In recent years, morphology has fallen behind data gathering, deposition and transparency practices considered as standard in other biological disciplines, such as proteomics or genomics [5]. Apart from its multiple potential applications, the dataset presented here is therefore also intended to serve as a catalyst for new approaches aimed at the large-scale deposition of digital morphological and anatomical information [6].

### Scanned specimens

The deposited dataset comprises 141 MRI scans from 98 representative extant sea urchin species. The scanned specimens were whole sub-adult or adult individuals ranging in diameter or length from 5–43 mm. Most of the specimens were obtained from museum collections, where they are preserved in ethanol for long-term storage. Some of the specimens were collected and fixed more than 135 years ago, while others were collected a few months prior to scanning. Additional file 1 gives a taxonomical list of the species analyzed and provides specimen data (Table S1).

### Data acquisition and processing

Basic information on the protocols used for specimen preparation, contrasting, and scanning are provided in Additional file 1, complemented by a description of each file type produced during the MRI experiments (Table S2). More specific information on sample preparation and equipment or the application of contrast agents and different scanning protocols has been published elsewhere [7]. The detailed acquisition and reconstruction parameters for each scan can be found in the MRI metadata files deposited online together with the raw and derived image data [8].

Forty-four two-dimensional (2D) and 97 three-dimensional (3D) scans were obtained using various high-field MRI scanners. Table 1 provides information on the scanning systems employed in this study. 2D MRI scans have a reduced voxel resolution in the third dimension (e.g., 50 × 50 × 200 μm), while 3D MRI scans are characterized by an isotropic voxel resolution (e.g., 40 × 40 × 40 μm). In various instances, specimens were scanned twice using the same scanning protocol, but once before and once after the application of a contrast agent (Magnevist, Bayer HealthCare, Leverkusen, Germany). The folders containing scans of contrasted specimens have been specifically labeled using the word 'Magnevist'.

**Table 1:** Overview of high-field MRI scanners (Bruker BioSpin MRI GmbH, Ettlingen, Germany) employed in this study

**Model**	**Location**	**Magnet**	**Resonance frequency**	**Gradient system**	**Gradient strength**	**Resonator**
7 T PharmaScan 70/16	Charité-Universitätsmedizin Berlin, Germany	Superconducting, 160 mm horizontal bore	^1^H, 300 MHz	Actively shielded, inner Ø 90 mm	300 mT/m	^1^H, linear volume resonator, inner Ø 38 mm
7 T BioSpec 70/20	Universität Würzburg, Germany	Superconducting, 200 mm horizontal bore	^1^H, 300 MHz	Actively shielded, inner Ø 90 mm	700 mT/m	^1^H, quadrature volume resonator, inner Ø 72 mm
9.4 T BioSpec 94/20	Universitätsklinikum Münster, Germany	Superconducting, 200 mm horizontal bore	^1^H, 400 MHz	Actively shielded, inner Ø 60 and 120 mm	720 mT/m and 1 T/m	^1^H, CryoProbe transmit-receive surface resonator and quadrature volume resonator, inner Ø 35 and 72 mm
9.4 T AVANCE 400WB	Leibniz-Institut für Molekulare Pharmakologie, Berlin, Germany	Superconducting, 89 mm vertical bore	^1^H, 400 MHz	Actively shielded, inner Ø 40 mm	1 T/m	^1^H, linear volume resonator, inner Ø 30 mm
17.6 T AVANCE 750WB	Universtität Würzburg, Germany	Superconducting, 89 mm vertical bore	^1^H, 750 MHz	Actively shielded, inner Ø 40 mm	1 T/m	^1^H, linear volume resonator, inner Ø 5 and 20 mm

For each scan, one image stack in tagged image file format (TIFF, .tif) was created based on the standard Bruker MRI 2dseq raw image file using the software ImageJ [9]. To facilitate a rapid recognition of internal structures, TIFF stacks based on 3D scans were rotated to a standardized orientation along the oral-aboral axis of the animal using the tool TransformJ Rotate, which is part of the ImageJ plugin TransformJ [10]. In addition, some of the TIFF stacks were reduced in their pixel dimensions by removing uninformative parts using the ‘Image:Crop’ command in ImageJ.

### Data quality

The suitability of a given specimen for MRI was ascertained through visual inspection of the MRI scout images and the low-resolution scans performed prior to scanning at high resolutions [7]. The achieved 2D and 3D scan resolutions constitute the current state-of-the-art in high-field MRI at the given fields of view and are largely comparable to results derived from dissections carried out under direct observation through a stereomicroscope [2]. The quality of a given scan depended on various factors, some of which were outside our control, such as specimen health prior to fixation, the fixation itself, or the quality of the long-term storage. Although the 141 deposited scans constitute a selection of those with the best quality, several scans still show a significant presence of artifacts. These artifacts are primarily related to the biology of the animal, in particular the presence of para- or ferromagnetic substances contained within the digestive tract [7]. Additional file 1 provides brief information on artifacts encountered in each scan.

### Potential uses

The methodological approach employed here allows conducting high-throughput analyses of hundreds or even thousands of zoological specimens [7]. Potential uses of the present dataset include morphometric or volumetric analyses of internal organs [11] and interactive exploration of sea urchin anatomy using digital 2D and 3D visualization tools [12]. For example, MRI scans with an isotropic voxel resolution are particularly suitable for 3D modeling [13]. In addition, MRI stacks can be aligned with image data derived from complementary non-invasive imaging techniques that permit visualizing mineralized structures, in particular micro-computed tomography [14]. Furthermore, the relatively quick 2D MRI scanning protocols used for some of the deposited scans could find application in the *in vivo* study of sea urchins whose gonads are intended for human consumption.

### Relevance of the dataset

The dataset presented here constitutes a representative sample of sea urchin structural diversity. No significant differences in scanning results were observed when employing freshly fixed or museum specimens [2], while the application of a contrast agent resulted in an improved signal-to-noise ratio, as well as a reduction of the negative effects of artifacts [7]. The MRI data allow identification of numerous internal organs, including lantern muscles [15], axial complex [16], gastric caecum [17], or intestinal caecum [18]. These studies demonstrate that initial difficulties with regard to the interpretation of MRI data do not render the scanning approach itself unsuitable [19].

Due to the digital nature of data based on non-invasive imaging techniques, the rapid online dissemination of morphological and anatomical information has finally become possible. This development is bound to lead to an unprecedented level of data availability and transparency in zoomorphology and paleontology, ultimately resulting in more widespread data mining in these two scientific fields [20]. Furthermore, the enforced deposition of digital morphological data as a prerequisite for publication will pave the way for the correlation of geno- and phenotype on a large scale [6]. We believe that digital datasets and enforced data deposition constitute essential components for the success of the expanding field of morphomics, which aims to complement the already established ‘omics’ disciplines [21].

## Availability of supporting data

The dataset supporting the results of this study is available in the GigaScience repository, *Giga*DB [8]. Additional file 1 provides specimen data, detailed information on data availability and requirements, as well as information on the preparation, contrasting and scanning of sea urchin specimens. The authors ask that any publication arising from the use of the deposited data acknowledges the source of the dataset. See [22] for a discussion of copyright licenses and waiver agreements used in open access research.

## Abbreviations

2D: Two-dimensional; 3D: Three-dimensional; MRI: Magnetic resonance imaging; TIFF: Tagged image file format.

## Competing interests

The authors declare that they have no competing interests.

## Authors’ contributions

AZ designed the study, drafted the manuscript, and gathered, analyzed and curated data. CF, SM, NN, and LS designed the experiments and gathered data. All authors read and approved the final manuscript.

## Supplementary Material

Additional file 1 **Supplementary specimen information and tables. Table S1.** Overview of the dataset comprising 141 MRI scans of 98 extant sea urchin species. **Table S2.** Overview of Bruker MRI file types. Click here for file
