# Supplementary material for: A dataset comprising 141 magnetic resonance imaging scans of 98 extant sea urchin species
Source: Gigascience. 2014 Oct 14;3:21. doi: 10.1186/2047-217X-3-21 (PMC4212584; doi:10.1186/2047-217X-3-21)
Supplement: Additional file 1 — Supplementary specimen information and tables. Table S1. Overview of the dataset comprising 141 MRI scans of 98 extant sea urchin species. Table S2. Overview of Bruker MRI file types. [file 2047-217X-3-21-S1.pdf]

Additional File 1. Supplementary specimen information and tables.

Table S1. Overview of the dataset comprising 141 MRI scans of 98 extant sea urchin species.

The artifacts mentioned in the last column have been classified according to the list of potential MRI artifacts provided in [1]. Explanations for the abbreviations used here are provided at the end of the table.

Family	Species	Scan name	Specimen (number; locality; size)	Experiment (scanner; method; parameters; resolution)	Artifacts
Histocidaridae Lambert, 1900	*Histocidaris elegans* (A. Agassiz, 1879)	3D_81_um	ZMH E307; 00°15' N 98°08' E, Nias Island, Indonesia, Indian Ocean, 614 m, Deutsche Tiefsee-Expedition Station 31; 24 mm	7 T PharmaScan; FLASH; TE 6.656 ms, TR 30 ms, NA 12, FOV (3.12 cm)^3, MTX (384 px)^3, TA 14 h 44 min 44 s; (81 µm)^3	Strong susceptibility artifacts caused by paramagnetic substance contained within the digestive tract
		3D_81_um_Magnevist	ZMH E307; 00°15' N 98°08' E, Nias Island, Indonesia, Indian Ocean, 614 m, Deutsche Tiefsee-Expedition Station 31; 24 mm	7 T PharmaScan; FLASH; TE 6.656 ms, TR 30 ms, NA 12, FOV (3.12 cm)^3, MTX (384 px)^3, TA 14 h 44 min 44 s; (81 µm)^3	Strong susceptibility artifacts caused by paramagnetic substance contained within the digestive tract
Ctenocidaridae Mortensen, 1928	*Ctenocidaris nutrix* (Thomson, 1876)	3D_79_um	BMNH 1956.10.5.1; Heard Island, Indian Ocean, 274 m, Challenger Expedition Station 150; 24 mm	7 T PharmaScan; FLASH; TE 6.656 ms, TR 30 ms, NA 14, FOV (3.05 cm)^3, MTX (384 px)^3, TA 17 h 12 min 11 s; (79 µm)^3	None
	Notocidaris gaussensis Mortensen, 1909	3D_79_um	ZMB 5456; Gauss Base, Antarctica, Southern Ocean, 350-385 m, Deutsche Südpolar-Expedition 1901-1903; 25 mm	7 T PharmaScan; FLASH; TE 6.656 ms, TR 30 ms, NA 16, FOV (3.05 cm)^3, MTX (384 px)^3, TA 19 h 39 min 38 s; (79 µm)^3	Local susceptibility artifacts caused by paramagnetic substance contained within the digestive tract
Cidaridae Gray, 1825	*Austrocidaris canaliculata* (A. Agassiz, 1863)	3D_79_um	ZMB 2244; 43°56' S 60°52' W, Argentina, Atlantic Ocean, 109 m, R/V Gazelle, February 1876; 21 mm	7 T PharmaScan; FLASH; TE 6.656 ms, TR 30 ms, NA 14, FOV (3.05 cm)^3, MTX (384 px)^3, TA 17 h 12 min 11 s; (79 µm)^3	Local susceptibility artifacts caused by paramagnetic substance contained within the digestive tract
	Cidaris cidaris (Linnaeus, 1758)	3D_81_um_Magnevist	BMNH 1925.10.30.103-113; Wyville Thomson Ridge,	7 T PharmaScan; FLASH; TE 6.656 ms, TR 30 ms, NA 12, FOV (3.12	Mild susceptibility and antialiasing artifacts

			Atlantic Ocean, 439 m, R/V Triton; 30 mm	cm)^3, MTX (384 px)^3, TA 14 h 44 min 44 s; (81 µm)^3	
	Eucidaris metularia (Lamarck, 1816)	3D_44_um_Magnevist	BMNH 1969.5.1.15-40; Aldabra, Indian Ocean, 1969; 16 mm	17.6 T AVANCE; GEFI; TE 2.573 ms, TR 20 ms, NA 52, FOV (1.7 cm)^3, MTX (512 px)^3, TA 42 h 35 min 54 s; (44 µm)^3	Strong radiofrequency field variation artifacts (?)
		3D_81_um_Magnevist	BMNH 1969.5.1.15-40; Aldabra, Indian Ocean, 1969; 20 mm	7 T PharmaScan; FLASH; TE 6.656 ms, TR 30 ms, NA 12, FOV (3.12 cm)^3, MTX (384 px)^3, TA 14 h 44 min 44 s; (81 µm)^3	None
	Eucidaris thouarsii (L. Agassiz & Desor, 1846)	2D_Magnevist	ZMB 1369; Panama, Pacific Ocean; 14 mm	9.4 T AVANCE; RARE; TE 11.59 ms, TR 2712.5 ms, NA 3, FOV 3 x 3 cm, MTX 600 x 600 px, ST 0.2 mm, NS 50, TA 10 min 10 s; 50 x 50 x 200 µm	Local susceptibility artifacts caused by paramagnetic substance contained within the digestive tract
	Eucidaris tribuloides (Lamarck, 1816)	2D_Magnevist	ZMB 5474; Strait between St. Thomas and St. John, Caribbean Sea, Kükenthal & Hartmeyer Westindienreise 1906-1907, January 1907; 24 mm	9.4 T AVANCE; RARE; TE 11.59 ms, TR 2712.5 ms, NA 3, FOV 3 x 3 cm, MTX 600 x 600 px, ST 0.2 mm, NS 50, TA 10 min 10 s; 50 x 50 x 200 µm	None
	Hesperocidaris panamensis (A. Agassiz, 1898)	2D_Magnevist	ZMB 5407; Panama, Pacific Ocean, 183 m; 21 mm	9.4 T AVANCE; RARE; TE 11.59 ms, TR 2712.5 ms, NA 3, FOV 3 x 3 cm, MTX 600 x 600 px, ST 0.2 mm, NS 50, TA 10 min 10 s; 50 x 50 x 200 µm	Local susceptibility artifacts caused by paramagnetic substance contained within the digestive tract
	Stereocidaris indica Döderlein, 1901	3D_79_um	ZMB 7364; 01°49' N 45°29' E, Mogadishu, Somalia, Indian Ocean, 1134 m, Deutsche Tiefsee-Expedition Station 256, March 1899; 28 mm	7 T PharmaScan; FLASH; TE 6.656 ms, TR 30 ms, NA 14, FOV (3.05 cm)^3, MTX (384 px)^3, TA 17 h 12 min 11 s; (79 µm)^3	Strong susceptibility artifacts caused by paramagnetic substance contained within the digestive tract
Psychocidaridae Ikeda, 1936	*Psychocidaris ohshimai* Ikeda, 1935	3D_79_um	NHMW 2010/0240/0001; Balicasag Island, Philippines, Pacific Ocean, 150-200 m; 28 mm	7 T PharmaScan; FLASH; TE 6.656 ms, TR 30 ms, NA 11, FOV (3.05 cm)^3, MTX (384 px)^3, TA 13 h 31 min 0 s; (79 µm)^3	Antialiasing artifact and strong susceptibility artifacts caused by paramagnetic substance contained within the digestive tract
Micropygidae Mortensen, 1903	*Micropyga tuberculata* A. Agassiz, 1879	3D_81_um	BMNH 98.8.8.45/6; Blanche Bay, Papua New Guinea, Pacific Ocean; 27 mm	7 T PharmaScan; FLASH; TE 6.656 ms, TR 30 ms, NA 12, FOV (3.12 cm)^3, MTX (384 px)^3, TA 14 h 44 min	Strong susceptibility artifacts caused by paramagnetic substance contained within the digestive

				44 s; (81 µm)^3	tract
		3D_81_um_Magnevist	BMNH 98.8.8.45/6; Blanche Bay, Papua New Guinea, Pacific Ocean; 27 mm	7 T PharmaScan; FLASH; TE 6.656 ms, TR 30 ms, NA 12, FOV (3.12 cm)^3, MTX (384 px)^3, TA 14 h 44 min 44 s; (81 µm)^3	Strong susceptibility artifacts caused by paramagnetic substance contained within the digestive tract
Diadematidae Gray, 1855	*Centrostephanus longispinus* (Philippi, 1845)	3D_66_um_Magnevist	BMNH 1952.3.26.64-8; Accra, Ghana, January 1951; 14 mm	17.6 T AVANCE; GEFI; TE 1.768 ms, TR 20 ms, NA 14, FOV 1.4 x 1.4 x 1.7 cm, MTX 212 x 212 x 256 px, TA 4 h 13 min 1 s; (66 µm)^3	Strong susceptibility artifacts caused by paramagnetic substance contained within the digestive tract
	Diadema antillarum Philippi, 1845	2D_Magnevist	ZMB 4374; Nassau, Bahamas, Caribbean Sea; 14 mm	9.4 T AVANCE; RARE; TE 11.59 ms, TR 2712.5 ms, NA 3, FOV 3 x 3 cm, MTX 600 x 600 px, ST 0.2 mm, NS 50, TA 10 min 10 s; 50 x 50 x 200 µm	Mild susceptibility artifacts caused by air bubbles and paramagnetic substance contained within the digestive tract
	Diadema savignyi (Audoiun, 1829)	3D_40_um_Magnevist	ZMB 7411; Red Sea, Indian Ocean, 2006; 19 mm	17.6 T AVANCE; GEFI; TE 2.84 ms, TR 20 ms, NA 22, FOV 1.7 x 1.7 x 1.4 cm, MTX 420 x 420 x 348 px, TA 17 h 51 min 50 s; (40 µm)^3	None
	Diadema setosum (Leske, 1778)	2D_Magnevist	ZMB 4814; Java Island, Indonesia, Indian Ocean; 18 mm	9.4 T AVANCE; RARE; TE 11.59 ms, TR 2712.5 ms, NA 3, FOV 3 x 3 cm, MTX 600 x 600 px, ST 0.2 mm, NS 50, TA 10 min 10 s; 50 x 50 x 200 µm	Strong susceptibility artifacts caused by air bubbles and paramagnetic substance contained within the digestive tract as well as aliasing and radiofrequency field variation artifacts
	Echinothrix diadema (Linnaeus, 1758)	2D_Magnevist	ZMB 2346; Mombasa, Kenya, Indian Ocean; 21 mm	9.4 T AVANCE; RARE; TE 11.59 ms, TR 2712.5 ms, NA 3, FOV 3 x 3 mm, MTX 600 x 600 px, ST 0.2 mm, NS 50, TA 10 min 10 s; 50 x 50 x 200 µm	Local susceptibility artifacts caused by paramagnetic substance contained within the digestive tract and air bubbles
Aspidodiadematidae Duncan, 1889	*Aspidodiadema hawaiiense* Mortensen, 1939	3D_81_um	NMNH 27588; Diamond Head, Oahu Island, Hawaii, USA, Pacific Ocean, 470-538 m, Hawaiian Explorations, R/V Albatross, May 1902; 24 mm	7 T PharmaScan; FLASH; TE 6.656 ms, TR 30 ms, NA 12, FOV (3.12 cm)^3, MTX (384 px)^3, TA 14 h 44 min 44 s; (81 µm)^3	Local susceptibility artifacts caused by paramagnetic substance contained within the digestive tract
		3D_81_um_Magnevist	NMNH 27588; Diamond Head, Oahu Island, Hawaii, USA, Pacific Ocean, 470-538	7 T PharmaScan; FLASH; TE 6.656 ms, TR 30 ms, NA 12, FOV (3.12 cm)^3, MTX (384 px)^3, TA 14 h 44 min	Local susceptibility artifacts caused by paramagnetic substance contained within the digestive

			m, Hawaiian Explorations, R/V Albatross, May 1902; 24 mm	44 s; (81 µm)^3	tract
	Aspidodiadema tonsum A. Agassiz, 1879	3D_60_um_Magnevist	NIWA 44818; 31°58'82'' S 174°15'87'' E, Three Kings Plateau, New Zealand, Pacific Ocean, 700 m, Station Z9026; 16 mm	9.4 T BioSpec; FLASH; TE 5.094 ms, TR 30 ms, NA 2, FOV (2.7 cm)^3, MTX (448 px)^3, TA 3 h 20 min 42 s; (60 µm)^3	Local susceptibility artifacts caused by paramagnetic substance contained within the digestive tract
	Plesiodiadema indicum (Döderlein, 1900)	3D_81_um	ZMB 7232; 00°15' N 98°07' E, Nias Island, Indonesia, Indian Ocean, Deutsche Tiefsee-Expedition Station 199, February 1899; 22 mm	7 T PharmaScan; FLASH; TE 6.656 ms, TR 30 ms, NA 12, FOV (3.12 cm)^3, MTX (384 px)^3, TA 14 h 44 min 44 s; (81 µm)^3	Strong susceptibility artifacts caused by paramagnetic substance contained within the digestive tract
		3D_81_m_Magnevist	ZMB 7232; 00°15' N 98°07' E, Nias Island, Indonesia, Indian Ocean, Deutsche Tiefsee-Expedition Station 199, February 1899; 22 mm	7 T PharmaScan; FLASH; TE 6.656 ms, TR 30 ms, NA 12, FOV (3.12 cm)^3, MTX (384 px)^3, TA 14 h 44 min 44 s; (81 µm)^3	Strong susceptibility artifacts caused by paramagnetic substance contained within the digestive tract
Pedinidae Pomel, 1883	*Caenopedina mirabilis* (Döderlein, 1885)	3D_81_um	NMNH 31182; 33°35'20'' N 135°10'50'' E, South off Shirahama, Honshu Island, Japan, Pacific Ocean, 349 m, Northwestern Pacific Expedition, R/V Albatross, August 1906; 15 mm	7 T PharmaScan; FLASH; TE 6.656 ms, TR 30 ms, NA 12, FOV (3.12 cm)^3, MTX (384 px)^3, TA 14 h 44 min 44 s; (81 µm)^3	Strong susceptibility artifacts caused by paramagnetic substance contained within the digestive tract
		3D_81_um_Magnevist	NMNH 31182; 33°35'20'' N 135°10'50'' E, South off Shirahama, Honshu Island, Japan, Pacific Ocean, 349 m, Northwestern Pacific Expedition, R/V Albatross, August 1906; 15 mm	7 T PharmaScan; FLASH; TE 6.656 ms, TR 30 ms, NA 12, FOV (3.12 cm)^3, MTX (384 px)^3, TA 14 h 44 min 44 s; (81 µm)^3	Strong susceptibility artifacts caused by paramagnetic substance contained within the digestive tract
Saleniidae L. Agassiz, 1838	*Salenia goesiana* Lovén, 1874	3D_81_um	NMNH 14581; N off Havana, Cuba, Caribbean Sea, R/V Albatross, 1886; 11 mm	7 T PharmaScan; FLASH; TE 6.656 ms, TR 30 ms, NA 12, FOV (3.12 cm)^3, MTX (384 px)^3, TA 14 h 44 min 44 s; (81 µm)^3	Strong susceptibility artifacts caused by air bubbles and paramagnetic substance contained within the digestive tract

		3D_81_um_Magnevist	NMNH 14581; N off Havana, Cuba, Caribbean Sea, R/V Albatross, 1886; 17 mm	7 T PharmaScan; FLASH; TE 6.656 ms, TR 30 ms, NA 12, FOV (3.12 cm)^3, MTX (384 px)^3, TA 14 h 44 min 44 s; (81 µm)^3	Mild susceptibility artifacts caused by air bubbles and paramagnetic substance contained within the digestive tract
	Salenocidaris hastigera (A. Agassiz, 1879)	3D_81_um	ZMB 5816; 00°02' S 73°24' E, Maldives, Indian Ocean, 2253 m, Deutsche Tiefsee-Expedition Station 219, 1898; 15 mm	7 T PharmaScan; FLASH; TE 6.656 ms, TR 30 ms, NA 12, FOV (3.12 cm)^3, MTX (384 px)^3, TA 14 h 44 min 44 s; (81 µm)^3	Mild susceptibility artifacts caused by air bubbles and paramagnetic substance contained within the digestive tract
		3D_81_um_Magnevist	ZMB 5816; 00°02' S 73°24' E, Maldives, Indian Ocean, 2253 m, Deutsche Tiefsee-Expedition Station 219, 1898; 15 mm	7 T PharmaScan; FLASH; TE 6.656 ms, TR 30 ms, NA 12, FOV (3.12 cm)^3, MTX (384 px)^3, TA 14 h 44 min 44 s; (81 µm)^3	Mild susceptibility artifacts caused by air bubbles and paramagnetic substance contained within the digestive tract
Stomopneustidae Mortensen, 1903	*Stomopneustes variolaris* (Lamarck, 1816)	2D_Magnevist	NMNH E45930; 02°50'56'' N 95°56'24'' E, Pulo Pemju, Greater Sunda Islands, Indonesia, Indian Ocean, R/V Te Vega, November 1963; 22 mm	9.4 T AVANCE; RARE; TE 11.59 ms, TR 2712.5 ms, NA 3, FOV 3 x 3 cm, MTX 600 x 600 px, ST 0.2 mm, NS 50, TA 10 min 10 s; 50 x 50 x 200 µm	Mild susceptibility artifacts caused by paramagnetic substance contained within the digestive tract
		3D_81_um	NMNH E45930; 02°50'56'' N 95°56'24'' E, Pulo Pemju, Greater Sunda Islands, Indonesia, Indian Ocean, R/V Te Vega, November 1963; 22 mm	7 T PharmaScan; FLASH; TE 6.656 ms, TR 30 ms, NA 12, FOV (3.12 cm)^3, MTX (384 px)^3, TA 14 h 44 min 44 s; (81 µm)^3	Strong susceptibility artifacts caused by air bubbles and paramagnetic substance contained within the digestive tract
		3D_81_um_Magnevist	NMNH E45930; 02°50'56'' N 95°56'24'' E, Pulo Pemju, Greater Sunda Islands, Indonesia, Indian Ocean, R/V Te Vega, November 1963; 22 mm	7 T PharmaScan; FLASH; TE 6.656 ms, TR 30 ms, NA 12, FOV (3.12 cm)^3, MTX (384 px)^3, TA 14 h 44 min 44 s; (81 µm)^3	Strong susceptibility artifacts caused by air bubbles and paramagnetic substance contained within the digestive tract
Glyptocidaridae Jensen, 1982	*Glyptocidaris crenularis* A. Agassiz, 1863	3D_90_um_Magnevist	ZSM 20011444; Bay of Funka, Hokkaido Island, Japan, Pacific Ocean, 1969; 43 mm	7 T BioSpec; FLASH; TE 2.88 ms, TR 30 ms, NA 28, FOV 3 x 4.6 x 4.6 cm, MTX 400 x 512 x 512 px, TA 61 h 10 min 0 s; (90 µm)^3	Strong susceptibility artifacts caused by air bubbles and paramagnetic substance contained within the digestive tract

Arbaciidae Gray, 1855	*Arbacia dufresnii* (Blainville, 1825)	2D_Magnevist	ZMB 2222; Tuesday Bay, Nodales Islands, Strait of Magellan, Pacific Ocean, 4.5 m, R/V Gazelle, February 1876; 23 mm	9.4 T AVANCE; RARE; TE 11.59 ms, TR 2712.5 ms, NA 3, FOV 3 x 3 cm, MTX 600 x 600 px, ST 0.2 mm, NS 60, TA 12 min 12 s; 50 x 50 x 200 µm	None
	Arbacia lixula (Linnaeus, 1758)	3D_44_um_Magnevist	BMNH 1952.3.26.31-36; Hospital Reef, Axim, Ghana, April 1949; 18 mm	17.6 T AVANCE; GEFI; TE 2.575 ms, TR 20 ms, NA 36, FOV 1.7 x 1.5 x 1.7 cm, MTX 384 x 340 x 384 px, TA 26 h 6 min 43 s; $(44\ \mu m)^3$	Strong radiofrequency field variation artifacts (?)
	Coelopleurus floridanus A. Agassiz, 1872	3D_60_um_Magnevist	PPSIO; Great Meteor Tablemount, Atlantic Ocean, R/V Ikhtiandr Cruise 8 Station 42, July 1982; 22 mm	9.4 T BioSpec; FLASH; TE 5.24 ms, TR 30 ms, NA 2, FOV $(2.8\ cm)^3$, MTX $(464\ px)^3$, TA 3 h 35 min 17 s; $(60\ \mu m)^3$	Strong susceptibility artifacts caused by paramagnetic substance contained within the digestive tract
	Podocidaris sp.	3D_31_um_Magnevist	PPSIO; 25°40'00'' S 86°46'05'' W, W off Chile, Pacific Ocean, 430-910 m, R/V Ikhtiandr Station N6, October 1980; 9 mm	9.4 T BioSpec; FLASH; TE 4.554 ms, TR 30 ms, NA 4, FOV 1.1 x 0.75 x 1.1 cm, MTX 360 x 242 x 360 px, TA 2 h 54 min 14 s; $(31\ \mu m)^3$	Strong susceptibility artifacts caused by paramagnetic substance contained within the digestive tract and air bubbles
	Tetrapygus niger (Molina, 1782)	2D_Magnevist	ZMB 1346; Cape Horn, Pacific Ocean; 16 mm	9.4 T AVANCE; RARE; TE 11.59 ms, TR 2712.5 ms, NA 3, FOV 3 x 3 cm, MTX 600 x 600 px, ST 0.2 mm, NS 50, TA 10 min 10 s; 50 x 50 x 200 µm	Strong movement artifact and mild susceptibility artifact caused by paramagnetic substance contained within the digestive tract
Parasaleniidae Mortensen, 1903	*Parasalenia gratiosa* A. Agassiz, 1863	2D_Magnevist	ZMB 6356; Madang, Papua New Guinea, Pacific Ocean; 25 mm	9.4 T AVANCE; RARE; TE 11.59 ms, TR 2712.5 ms, NA 3, FOV 3 x 3 cm, MTX 600 x 600 px, ST 0.2 mm, NS 50, TA 10 min 10 s; 50 x 50 x 200 µm	Local susceptibility artifacts caused by paramagnetic substance contained within the digestive tract and air bubble
		3D_79_um	BMNH 1983.2.15.7; Fisheries Research Station, Starfish Bay, Hong Kong, Pacific Ocean, 2 m, November 1977; 26 mm	7 T PharmaScan; FLASH; TE 6.656 ms, TR 30 ms, NA 15, FOV $(3.05\ cm)^3$, MTX $(384\ px)^3$, TA 17 h 12 min 11 s; $(79\ \mu m)^3$	Local susceptibility artifacts caused by paramagnetic substance contained within the digestive tract
Temnopleuridae A. Agassiz, 1872	*Amblypneustes pallidus* (Lamarck, 1816)	2D_Magnevist	ZMB 6334; Koombana Bay, Bunbury, Australia, Indian Ocean, 14-18 m, Südwest-Australienreise Station 56, July 1905; 22 mm	9.4 T AVANCE; RARE; TE 11.59 ms, TR 2712.5 ms, NA 3, FOV 3 x 3 cm, MTX 600 x 600 px, ST 0.2 mm, NS 100, TA 20 min 20 s; 50 x 50 x 200 µm	Mild antialiasing artifacts

	Holopneustes inflatus (A. Agassiz, 1872)	2D_Magnevist	ZMB 2639; Port Jackson, Sydney, Australia, Pacific Ocean; 24 mm	9.4 T AVANCE; RARE; TE 11.59 ms, TR 2712.5 ms, NA 3, FOV 3 x 3 cm, MTX 600 x 600 px, ST 0.2 mm, NS 90, TA 18 min 18 s; 50 x 50 x 200 µm	None
	Mespilia globulus (Linnaeus, 1758)	3D_36_um_Magnevist	ZMB 5620; Madang, Papua New Guinea, Pacific Ocean, 1909; 13 mm	17.6 T AVANCE; GEFI; TE 2.65 ms, TR 20 ms, NA 40, FOV $(1.4\ cm)^3$, MTX $(384\ px)^3$, TA 32 h 46 min 4 s; $(36\ µm)^3$	None
		3D_81_um	ZMB 5620; Madang, Papua New Guinea, Pacific Ocean, 1909; 20 mm	7 T PharmaScan; FLASH; TE 6.656 ms, TR 30 ms, NA 12, FOV $(3.12\ cm)^3$, MTX $(384\ px)^3$, TA 14 h 44 min 44 s; $(81\ µm)^3$	Strong susceptibility artifacts caused by paramagnetic substance contained within the digestive tract
	Pseudechinus magellanicus (Philippi, 1857)	2D_Magnevist	ZMB 2188; Punta Arenas, Chile, Pacific Ocean, 2-4 m, R/V Gazelle, February 1876; 21 mm	9.4 T AVANCE; RARE; TE 11.59 ms, TR 2712.5 ms, NA 3, FOV 3 x 3 cm, MTX 600 x 600 px, ST 0.2 mm, NS 60, TA 12 min 12 s; 50 x 50 x 200 µm	Mild susceptibility artifacts caused by paramagnetic substance contained within the digestive tract
	Salmacis sphaeroides (Linnaeus, 1758)	2D_Magnevist	ZMB 4337; Gulf of Thailand, South China Sea, Pacific Ocean, 37 m; 16 mm	9.4 T AVANCE; RARE; TE 11.59 ms, TR 2712.5 ms, NA 3, FOV 3 x 3 cm, MTX 600 x 600 px, ST 0.2 mm, NS 50, TA 10 min 10 s; 50 x 50 x 200 µm	Strong movement artifact
	Temnopleurus michaelseni (Döderlein, 1914)	2D_Magnevist	ZMB 6331; Rottnest Island, Australia, Indian Ocean, Südwest-Australienreise Station 46, September 1905; 14 mm	9.4 T AVANCE; RARE; TE 11.59 ms, TR 2712.5 ms, NA 3, FOV 3 x 3 cm, MTX 600 x 600 px, ST 0.2 mm, NS 50, TA 10 min 10 s; 50 x 50 x 200 µm	Mild movement artifact
	Temnopleurus reevesii (Gray, 1855)	2D_Magnevist	ZMB 3588; Tango, Japan, Pacific Ocean, March 1894; 18 mm	9.4 T AVANCE; RARE; TE 11.59 ms, TR 2712.5 ms, NA 3, FOV 3 x 3 cm, MTX 600 x 600 px, ST 0.2 mm, NS 50, TA 10 min 10 s; 50 x 50 x 200 µm	Strong susceptibility artifacts caused by paramagnetic substance contained within the digestive tract
	Temnopleurus toreumaticus (Leske, 1778)	2D_Magnevist	ZMB 2802; NE coast of Japan, Pacific Ocean; 24 mm	9.4 T AVANCE; RARE; TE 11.59 ms, TR 2712.5 ms, NA 3, FOV 3 x 3 cm, MTX 600 x 600 px, ST 0.2 mm, NS 60, TA 12 min 12 s; 50 x 50 x 200 µm	Mild susceptibility artifacts caused by paramagnetic substance contained within the digestive tract
Trigonocidaridae Mortensen, 1903	*Genocidaris maculata* A. Agassiz, 1869	3D_36_um_Magnevist	ZMB 5827; 06°19' S 12°02' E, mouth of Kongo river, Atlantic Ocean, 44 m,	17.6 T AVANCE; GEFI; TE 1.919 ms, TR 20 ms, NA 24, FOV $(0.7\ cm)^3$, MTX $(512\ px)^3$, TA 15 h 7 min 19 s;	Strong susceptibility artifacts caused by paramagnetic substance contained within the digestive

			Deutsche Tiefsee-Expedition Station 71; 8 mm	(36 µm)^3	tract
	Trigonocidaris albida A. Agassiz, 1869	3D_32_um_Magnevist	ZSM 20012468; 30°02'09'' N 28°24'02'' W, Great Meteor Tablemount, Atlantic Ocean, 326-332 m, R/V Meteor, July 1967; 15 mm	17.6 T AVANCE; GEFI; TE 2.224 ms, TR 20 ms, NA 38, FOV (0.8 cm)^3, MTX (512 px)^3, TA 15 h 33 min 53 s; (32 µm)^3	Mild susceptibility artifacts caused by paramagnetic substance contained within the digestive tract
Echinidae Gray, 1825	*Echinus esculentus* Linnaeus, 1758	2D_Magnevist	ZMB 3826; North Sea, 1896; 25 mm	9.4 T AVANCE; RARE; TE 11.59 ms, TR 2712.5 ms, NA 3, FOV 3 x 3 cm, MTX 600 x 600 px, ST 0.2 mm, NS 50, TA 10 min 10 s; 50 x 50 x 200 µm	Local susceptibility artifacts caused by paramagnetic substance contained within the digestive tract
		3D_81_um	ZMB 3826; North Sea, 1896; 25 mm	7 T PharmaScan; FLASH; TE 6.656 ms, TR 30 ms, NA 12, FOV (3.12 cm)^3, MTX (384 px)^3, TA 14 h 44 min 44 s; (81 µm)^3	Local susceptibility artifacts caused by paramagnetic substance contained within the digestive tract
		3D_81_um_Magenvist	ZMB 3826; North Sea, 1896; 25 mm	7 T PharmaScan; FLASH; TE 6.656 ms, TR 30 ms, NA 12, FOV (3.12 cm)^3, MTX (384 px)^3, TA 14 h 44 min 44 s; (81 µm)^3	Local susceptibility artifacts caused by paramagnetic substance contained within the digestive tract
	Gracilechinus acutus (Lamarck, 1816)	2D_Magnevist	ZMB 3714; 33°56' N 26°43' E, SE off Crete, Greece, Mediterranean Sea, 620 m, R/V Pola; 16 mm	9.4 T AVANCE; RARE; TE 11.59 ms, TR 2712.5 ms, NA 3, FOV 3 x 3 cm, MTX 600 x 600 px, ST 0.2 mm, NS 50, TA 10 min 10 s; 50 x 50 x 200 µm	Strong susceptibility artifacts caused by paramagnetic substance contained within the digestive tract and antialising artifact
	Gracilechinus alexandri (Danielssen & Koren, 1883)	2D_Magnevist	ZMB 4340; 61°42' N 09°36' W, W off Faroer Islands, Atlantic Ocean, 997 m, Ingolf Expedition Station 44; 19 mm	9.4 T AVANCE; RARE; TE 11.59 ms, TR 2712.5 ms, NA 3, FOV 3 x 3 cm, MTX 600 x 600 px, ST 0.2 mm, NS 50, TA 10 min 10 s; 50 x 50 x 200 µm	Strong susceptibility artifacts caused by paramagnetic substance contained within the digestive tract
	Polyechinus agulhensis (Döderlein, 1905)	2D_Magnevist	ZMB 7219; 35°10' S 23°02' E, W off Grue Bank, South Africa, Indian Ocean, November 1898; 23 mm	9.4 T AVANCE; RARE; TE 11.59 ms, TR 2712.5 ms, NA 3, FOV 3 x 3 cm, MTX 600 x 600 px, ST 0.2 mm, NS 50, TA 10 min 10 s; 50 x 50 x 200 µm	Local susceptibility artifacts caused by paramagnetic substance contained within the digestive tract
	Sterechinus agassizii Mortensen, 1910	2D_Magnevist	BMNH 1914.8.12.126-127; British Antarctic Expedition Station 355; 18 mm	9.4 T AVANCE; RARE; TE 11.59 ms, TR 2712.5 ms, NA 3, FOV 3 x 3 cm, MTX 600 x 600 px, ST 0.2 mm, NS 50, TA 10 min 10 s; 50 x 50 x 200 µm	Mild susceptibility artifacts caused by paramagnetic substance contained within the digestive tract
		3D_79_um	BMNH 1914.8.12.126-127;	7 T PharmaScan; FLASH; TE 6.656	Strong susceptibility artifacts

			British Antarctic Expedition Station 355; 18 mm	ms, TR 30 ms, NA 11, FOV (3.05 cm)^3, MTX (384 px)^3, TA 13 h 31 min 0 s; (79 µm)^3	caused by paramagnetic substance contained within the digestive tract
	Sterechinus antarcticus Koehler, 1901	2D_Magnevist	ZMB 5439; Gauss Base, Antarctica, Southern Ocean, 350-385 m, Deutsche Südpolar-Expedition 1901-1903; 23 mm	9.4 T AVANCE; RARE; TE 11.59 ms, TR 2712.5 ms, NA 3, FOV 3 x 3 cm, MTX 600 x 600 px, ST 0.2 mm, NS 50, TA 10 min 10 s; 50 x 50 x 200 µm	Strong susceptibility artifacts caused by paramagnetic substance contained within the digestive tract
	Sterechinus neumayeri (Meissner, 1900)	2D_Magnevist	ZMB 5442; Gauss Base, Antarctica, Southern Ocean, 350-385 m, Deutsche Südpolar-Expedition 1901-1903; 25 mm	9.4 T AVANCE; RARE; TE 11.59 ms, TR 2712.5 ms, NA 3, FOV 3 x 3 cm, MTX 600 x 600 px, ST 0.2 mm, NS 50, TA 10 min 10 s; 50 x 50 x 200 µm	Antialiasing artifact and mild susceptibility artifacts caused by paramagnetic substance contained within the digestive tract
Parechinidae Mortensen, 1903	*Loxechinus albus* (Molina, 1782)	2D_Magnevist	BMNH 1966.9.27.35; 49°09'52'' S 74°22'08'' W, S Chile, Pacific Ocean, Royal Society Expedition Station 39, December 1958; 27 mm	9.4 T AVANCE; RARE; TE 11.59 ms, TR 2712.5 ms, NA 3, FOV 3 x 3 cm, MTX 600 x 600 px, ST 0.2 mm, NS 50, TA 10 min 10 s; 50 x 50 x 200 µm	Local susceptibility artifact caused by air bubble
	Paracentrotus lividus (Lamarck, 1816)	2D_Magnevist	ZMB 7406; Costa Brava, Spain, Mediterranean Sea, 1 m, 2006; 25 mm	9.4 T AVANCE; RARE; TE 11.59 ms, TR 2712.5 ms, NA 3, FOV 3 x 3 cm, MTX 600 x 600 px, ST 0.2 mm, NS 50, TA 10 min 10 s; 50 x 50 x 200 µm	Strong artifact caused by radiofrequency field variations
		3D_81_um	ZMB 7406; Costa Brava, Spain, Mediterranean Sea, 1 m, 2006; 25 mm	7 T PharmaScan; FLASH; TE 6.656 ms, TR 30 ms, NA 12, FOV (3.12 cm)^3, MTX (384 px)^3, TA 14 h 44 min 44 s; (81 µm)^3	Local susceptibility artifacts caused by paramagnetic substance contained within the digestive tract
		3D_81_um_Magnevist	ZMB 7406; Costa Brava, Spain, Mediterranean Sea, 1 m, 2006; 25 mm	7 T PharmaScan; FLASH; TE 6.656 ms, TR 30 ms, NA 12, FOV (3.12 cm)^3, MTX (384 px)^3, TA 14 h 44 min 44 s; (81 µm)^3	Local susceptibility artifacts caused by paramagnetic substance contained within the digestive tract
	Parechinus angulosus (Leske, 1778)	2D_Magnevist	ZMB 5644; Angra Pequena, Lüderitz, Namibia, Atlantic Ocean, July 1903; 24 mm	9.4 T AVANCE; RARE; TE 11.59 ms, TR 2712.5 ms, NA 3, FOV 3 x 3 cm, MTX 600 x 600 px, ST 0.2 mm, NS 50, TA 10 min 10 s; 50 x 50 x 200 µm	Local susceptibility artifacts caused by paramagnetic substance contained within the digestive tract
	Psammechinus microtuberculatus	2D_Magnevist	ZMB 4770; Rovinj, Croatia, Mediterranean Sea, August	9.4 T AVANCE; RARE; TE 11.59 ms, TR 2712.5 ms, NA 3, FOV 3 x 3 cm,	Local susceptibility artifacts caused by paramagnetic substance

	(Blainville, 1825)		1902; 22 mm	MTX 600 x 600 px, ST 0.2 mm, NS 50, TA 10 min 10 s; 50 x 50 x 200 µm	contained within the digestive tract
	Psammechinus miliaris (P. L. S. Müller, 1771)	3D_44_um_Magnevist	Private collection; off Heligoland, North Sea, 2006; 17 mm	17.6 T AVANCE; GEFI; TE 2.97 ms, TR 20 ms, NA 40, FOV 1.7 x 1.4 x 1.7 cm, MTX 384 x 320 x 384 px, TA 15 h 1 min 7 s; $(44\ \mu m)^3$	Local susceptibility artifacts caused by paramagnetic substance contained within the digestive tract
		3D_81_um	ZMB 2011; Arendal, Norway, Skagerrak, North Sea, R/V Pommerania; 27 mm	7 T PharmaScan; FLASH; TE 6.656 ms, TR 30 ms, NA 12, FOV $(3.12\ cm)^3$, MTX $(384\ px)^3$, TA 14 h 44 min 44 s; $(81\ \mu m)^3$	Local susceptibility artifacts caused by paramagnetic substance contained within the digestive tract
		3D_81_um_Magnevist	ZMB 2011; Arendal, Norway, Skagerrak, North Sea, R/V Pommerania; 27 mm	7 T PharmaScan; FLASH; TE 6.656 ms, TR 30 ms, NA 12, FOV $(3.12\ cm)^3$, MTX $(384\ px)^3$, TA 14 h 44 min 44 s; $(81\ \mu m)^3$	Local susceptibility artifacts caused by paramagnetic substance contained within the digestive tract
Toxopneustidae Troschel, 1872	*Gymnechinus robillardi* (de Loriol, 1883)	2D_Magnevist	BMNH 1890.6.27.5-8; Sri Lanka, Indian Ocean; 22 mm	9.4 T AVANCE; RARE; TE 11.59 ms, TR 2712.5 ms, NA 3, FOV 3 x 3 cm, MTX 600 x 600 px, ST 0.2 mm, NS 50, TA 10 min 10 s; 50 x 50 x 200 µm	Mild susceptibility artifacts caused by paramagnetic substance contained within the digestive tract
		3D_79_um	BMNH 1890.6.27.5-8; Sri Lanka, Indian Ocean; 22 mm	7 T PharmaScan; FLASH; TE 6.656 ms, TR 30 ms, NA 14, FOV $(3.05\ cm)^3$, MTX $(384\ px)^3$, TA 17 h 12 min 11 s; $(79\ \mu m)^3$	Mild susceptibility artifacts caused by paramagnetic substance contained within the digestive tract
	Lytechinus variegatus (Lamarck, 1816)	2D_Magnevist	ZMB 5517; St. Thomas, Caribbean Sea, Kükenthal & Hartmeyer Westindienreise 1906-1907, July 1906; 19 mm	9.4 T AVANCE; RARE; TE 11.59 ms, TR 2712.5 ms, NA 3, FOV 3 x 3 cm, MTX 600 x 600 px, ST 0.2 mm, NS 50, TA 10 min 10 s; 50 x 50 x 200 µm	None
		3D_81_um	Private collection; Caribbean Sea; 27 mm	7 T PharmaScan; FLASH; TE 6.656 ms, TR 30 ms, NA 12, FOV $(3.12\ cm)^3$, MTX $(384\ px)^3$, TA 14 h 44 min 44 s; $(81\ \mu m)^3$	None
		3D_81_um_Magnevist	Private collection; Caribbean Sea; 27 mm	7 T PharmaScan; FLASH; TE 6.656 ms, TR 30 ms, NA 12, FOV $(3.12\ cm)^3$, MTX $(384\ px)^3$, TA 14 h 44 min 44 s; $(81\ \mu m)^3$	None
	Nudechinus scotiopremnus H.	2D_Magnevist	ZMB 6130; Golf of Suez, Red Sea, Indian Ocean; 23 mm	9.4 T AVANCE; RARE; TE 11.59 ms, TR 2712.5 ms, NA 3, FOV 3 x 3 cm,	Mild artifacts caused by radiofrequency field variations

	L. Clark, 1912			MTX 600 x 600 px, ST 0.2 mm, NS 50, TA 10 min 10 s; 50 x 50 x 200 µm	
	Sphaerechinus granularis (Lamarck, 1816)	2D_Magnevist	ZMB 2934; Azores, Portugal, Atlantic Ocean; 21 mm	9.4 T AVANCE; RARE; TE 11.59 ms, TR 2712.5 ms, NA 3, FOV 3 x 3 cm, MTX 600 x 600 px, ST 0.2 mm, NS 50, TA 10 min 10 s; 50 x 50 x 200 µm	Strong susceptibility artifacts caused by paramagnetic substance contained within the digestive tract
		3D_81_um	ZMB 2366; Madeira, Portugal, Atlantic Ocean, Langerhans Expedition; 26 mm	7 T PharmaScan; FLASH; TE 6.656 ms, TR 30 ms, NA 12, FOV (3.12 cm)^3, MTX (384 px)^3, TA 14 h 44 min 44 s; (81 µm)^3	Mild susceptibility artifacts caused by paramagnetic substance contained within the digestive tract
	Toxopneustes pileolus (Lamarck, 1816)	2D_Magnevist	ZMB 3871; Ralum, East New Britain, Papua New Guinea, Pacific Ocean; 15 mm	9.4 T AVANCE; RARE; TE 11.59 ms, TR 2712.5 ms, NA 3, FOV 3 x 3 cm, MTX 600 x 600 px, ST 0.2 mm, NS 50, TA 10 min 10 s; 50 x 50 x 200 µm	Strong susceptibility artifacts caused by paramagnetic substance contained within the digestive tract
	Tripneustes ventricosus (Lamarck, 1816)	2D_Magnevist	ZMB 5498; Hastings, Barbados, Caribbean Sea, Kükenthal & Hartmeyer Westindienreise 1906-1907; 24 mm	9.4 T AVANCE; RARE; TE 11.59 ms, TR 2712.5 ms, NA 3, FOV 3 x 3 cm, MTX 600 x 600 px, ST 0.2 mm, NS 50, TA 10 min 10 s; 50 x 50 x 200 µm	Mild susceptibility artifacts caused by paramagnetic substance contained within the digestive tract
Strongylocentrotidae Gregory, 1900	*Hemicentrotus pulcherrimus* (A. Agassiz, 1863)	2D_Magnevist	ZMB 6425; Kobe, Japan, Pacific Ocean; 27 mm	9.4 T AVANCE; RARE; TE 11.59 ms, TR 2712.5 ms, NA 3, FOV 3 x 3 cm, MTX 600 x 600 px, ST 0.2 mm, NS 50, TA 10 min 10 s; 50 x 50 x 200 µm	Strong antialiasing artifact and susceptibility artifacts caused by paramagnetic substance contained within the digestive tract and
	Pseudocentrotus depressus (A. Agassiz, 1863)	2D_Magnevist	ZMB 6426; Kobe, Japan, Pacific Ocean; 26 mm	9.4 T AVANCE; RARE; TE 11.59 ms, TR 2712.5 ms, NA 3, FOV 3 x 3 cm, MTX 600 x 600 px, ST 0.2 mm, NS 50, TA 10 min 10 s; 50 x 50 x 200 µm	Strong susceptibility artifacts caused by paramagnetic substance contained within the digestive tract
	Strongylocentrotus droebachiensis (O. F. Müller, 1776)	2D_Magnevist	ZMB 4422; Abeloya, Kong Karls Land, 40 m, Helgoland Expedition Station 32, 1898; 23 mm	9.4 T AVANCE; RARE; TE 11.59 ms, TR 2712.5 ms, NA 3, FOV 3 x 3 cm, MTX 600 x 600 px, ST 0.2 mm, NS 50, TA 10 min 10 s; 50 x 50 x 200 µm	Antialiasing artifact and mild susceptibility artifacts caused by paramagnetic substance contained within the digestive tract
	Strongylocentrotus purpuratus (Stimpson, 1857)	3D_42_um_Magnevist	CASIZ 5724; Morro Bay, San Luis Obispo, California, USA, September 1972; 17 mm	17.6 T AVANCE; GEFI; TE 2.502 ms, TR 20 ms, NA 32, FOV 1.5 x 1.4 x 1.6 cm, MTX 360 x 336 x 384 px, TA 22 h 56 min 15 s; (42 µm)^3	Mild susceptibility artifacts caused by paramagnetic substance contained within the digestive tract
		3D_81_um	CASIZ 5724; Morro Bay, San	7 T PharmaScan; FLASH; TE 6.656	None

			Luis Obispo, California, USA, September 1972; 23 mm	ms, TR 30 ms, NA 12, FOV (3.12 cm)^3, MTX (384 px)^3, TA 14 h 44 min 44 s; (81 µm)^3	
		3D_81_um_Magnevist	CASIZ 5724; Morro Bay, San Luis Obispo, California, USA, September 1972; 23 mm	7 T PharmaScan; FLASH; TE 6.656 ms, TR 30 ms, NA 12, FOV (3.12 cm)^3, MTX (384 px)^3, TA 14 h 44 min 44 s; (81 µm)^3	None
Echinometridae Gray, 1855	*Caenocentrotus gibbosus* (L. Agassiz in L. Agassiz & Desor, 1846)	2D_Magnevist	ZMB 5405; Paita, Peru, Pacific Ocean; 25 mm	9.4 T AVANCE; RARE; TE 11.59 ms, TR 2712.5 ms, NA 3, FOV 3 x 3 cm, MTX 600 x 600 px, ST 0.2 mm, NS 50, TA 10 min 10 s; 50 x 50 x 200 µm	Strong antialising artifact and local susceptibility artifacts caused by paramagnetic substance contained within the digestive tract
	Colobocentrotus atratus (Linnaeus, 1758)	2D_Magnevist	ZMB 4985; Jakarta, Indonesia, Pacific Ocean; 25 mm	9.4 T AVANCE; RARE; TE 11.59 ms, TR 2712.5 ms, NA 3, FOV 3 x 3 cm, MTX 600 x 600 px, ST 0.2 mm, NS 50, TA 10 min 10 s; 50 x 50 x 200 µm	Strong susceptibility artifacts caused by paramagnetic substance contained within the digestive tract
	Echinometra mathaei (Blainville, 1825)	3D_81_um	BMNH 1969.5.1.61-75; Aldabra, Indian Ocean, 1969; 25 mm	7 T PharmaScan; FLASH; TE 6.656 ms, TR 30 ms, NA 12, FOV (3.12 cm)^3, MTX (384 px)^3, TA 14 h 44 min 44 s; (81 µm)^3	None
		3D_81_um_Magnevist	BMNH 1969.5.1.61-75; Aldabra, Indian Ocean, 1969; 25 mm	7 T PharmaScan; FLASH; TE 6.656 ms, TR 30 ms, NA 12, FOV (3.12 cm)^3, MTX (384 px)^3, TA 14 h 44 min 44 s; (81 µm)^3	None
	Echinometra viridis A. Agassiz, 1863	2D_Magnevist	ZMB 5503; Dry Tortugas, Florida, USA, Gulf of Mexico, Kükenthal & Hartmeyer Westindienreise 1906-1907; 21 mm	9.4 T AVANCE; RARE; TE 11.59 ms, TR 2712.5 ms, NA 3, FOV 3 x 3 cm, MTX 600 x 600 px, ST 0.2 mm, NS 50, TA 10 min 10 s; 50 x 50 x 200 µm	Strong radiofrequency field variation artifacts (?)
	Echinostrephus molaris (Blainville, 1825)	2D_Magnevist	ZMB 4000; Mombasa, Kenya, Indian Ocean; 16 mm	9.4 T AVANCE; RARE; TE 11.59 ms, TR 2712.5 ms, NA 3, FOV 3 x 3 cm, MTX 600 x 600 px, ST 0.2 mm, NS 50, TA 10 min 10 s; 50 x 50 x 200 µm	None
	Heliocidaris crassispina (A. Agassiz, 1863)	2D_Magnevist	ZMB 6424; Kobe, Japan, Pacific Ocean; 20 mm	9.4 T AVANCE; RARE; TE 11.59 ms, TR 2712.5 ms, NA 3, FOV 3 x 3 cm, MTX 600 x 600 px, ST 0.2 mm, NS	None

				50, TA 10 min 10 s; 50 x 50 x 200 µm	
	Heliocidaris erythrogramma (Valenciennes, 1846)	2D_Magnevist	ZMB 5745; Port Jackson, Sydney, Australia, Pacific Ocean; 22 mm	9.4 T AVANCE; RARE; TE 11.59 ms, TR 2712.5 ms, NA 3, FOV 3 x 3 cm, MTX 600 x 600 px, ST 0.2 mm, NS 50, TA 10 min 10 s; 50 x 50 x 200 µm	None
	Heterocentrotus mammillatus (Linnaeus, 1758)	2D_Magnevist	ZMB 1567; Papua New Guinea, Pacific Ocean; 17 mm	9.4 T AVANCE; RARE; TE 11.59 ms, TR 2712.5 ms, NA 3, FOV 3 x 3 cm, MTX 600 x 600 px, ST 0.2 mm, NS 50, TA 10 min 10 s; 50 x 50 x 200 µm	Local susceptibility artifacts caused by paramagnetic substance contained within the digestive tract
Echinoneidae L. Agassiz & Desor, 1847	*Echinoneus cyclostomus* Leske, 1778	3D_59_um_Magnevist	BMNH 1969.5.1.105; Aldabra, Indian Ocean, 1969; 29 mm	17.6 T AVANCE; GEFI; TE 6.77 ms, TR 25 ms, NA 10, FOV 3 x 2.8 x 2.3 cm, MTX 512 x 480 x 400 px, TA 13 h 20 min; $(59\ \mu m)^3$	Local susceptibility artifact caused by air bubble
		3D_86_um	BMNH 1969.5.1.105; Aldabra, Indian Ocean, 1969; 34 mm	7 T PharmaScan; FLASH; TE 6.656 ms, TR 30 ms, NA 12, FOV $(3.3\ cm)^3$, MTX $(384\ px)^3$, TA 14 h 44 min 44 s; $(86\ \mu m)^3$	None
		3D_86_um_Magnevist	BMNH 1969.5.1.105; Aldabra, Indian Ocean, 1969; 34 mm	7 T PharmaScan; FLASH; TE 6.656 ms, TR 30 ms, NA 12, FOV $(3.3\ cm)^3$, MTX $(384\ px)^3$, TA 14 h 44 min 44 s; $(86\ \mu m)^3$	None
Cassidulidae L. Agassiz & Desor, 1847	*Cassidulus caribaearum* Lamarck, 1801	3D_81_um	CASIZ 112632; Loblolly Bay, Anegada, British Virgin Islands, Caribbean Sea, 0.5-1 m, April 1986; 19 mm	7 T PharmaScan; FLASH; TE 6.656 ms, TR 30 ms, NA 12, FOV $(3.12\ cm)^3$, MTX $(384\ px)^3$, TA 14 h 44 min 44 s; $(81\ \mu m)^3$	Mild susceptibility artifacts caused by paramagnetic substance contained within the digestive tract
		3D_81_um_Magnevist	CASIZ 112632; Loblolly Bay, Anegada, British Virgin Islands, Caribbean Sea, 0.5-1 m, April 1986; 19 mm	7 T PharmaScan; FLASH; TE 6.656 ms, TR 30 ms, NA 12, FOV $(3.12\ cm)^3$, MTX $(384\ px)^3$, TA 14 h 44 min 44 s; $(81\ \mu m)^3$	Mild susceptibility artifacts caused by paramagnetic substance contained within the digestive tract
Echinolampadidae Gray, 1851	*Echinolampas depressa* Gray, 1851	3D_81_um	NMNH E32955; 26°16'50'' N 83°23'49'' W, off Florida, USA, Gulf of Mexico, 55.5 m, SOFLA Expedition, May 1981; 28 mm	7 T PharmaScan; FLASH; TE 6.656 ms, TR 30 ms, NA 12, FOV $(3.12\ cm)^3$, MTX $(384\ px)^3$, TA 14 h 44 min 44 s; $(81\ \mu m)^3$	Mild susceptibility artifacts caused by paramagnetic substance contained within the digestive tract
		3D_81_um_Magnevist	NMNH E32955; 26°16'50'' N 83°23'49'' W, off Florida,	7 T PharmaScan; FLASH; TE 6.656 ms, TR 30 ms, NA 12, FOV (3.12	Mild susceptibility artifacts caused by paramagnetic substance

			USA, Gulf of Mexico, 55.5 m, SOFLA Expedition, May 1981; 28 mm	cm)^3, MTX (384 px)^3, TA 14 h 44 min 44 s; (81 µm)^3	contained within the digestive tract
Clypeasteridae L. Agassiz, 1835	*Arachnoides placenta* (Linnaeus, 1758)	3D_81_um	ZMB 1439; Atapupu, Timor, Indonesia, Indian Ocean, R/V Gazelle, 1899; 30 mm	7 T PharmaScan; FLASH; TE 6.656 ms, TR 30 ms, NA 12, FOV (3.12 cm)^3, MTX (384 px)^3, TA 14 h 44 min 44 s; (81 µm)^3	Mild susceptibility artifacts caused by air bubbles and paramagnetic substance contained within the digestive tract
	Clypeaster reticulatus (Linnaeus, 1758)	3D_81_um	NMNH 34282; Penguin Bank, Oahu Island, Hawaii, USA, Pacific Ocean, 49-53 m, R/V Albatross, July 1902; 26 mm	7 T PharmaScan; FLASH; TE 6.656 ms, TR 30 ms, NA 12, FOV (3.12 cm)^3, MTX (384 px)^3, TA 14 h 44 min 44 s; (81 µm)^3	Local susceptibility artifact caused by air bubble
		3D_81_um_Magnevist	NMNH 34282; Penguin Bank, Oahu Island, Hawaii, USA, Pacific Ocean, 49-53 m, R/V Albatross, July 1902; 26 mm	7 T PharmaScan; FLASH; TE 6.656 ms, TR 30 ms, NA 12, FOV (3.12 cm)^3, MTX (384 px)^3, TA 14 h 44 min 44 s; (81 µm)^3	Local susceptibility artifact caused by air bubble
	Clypeaster rosaceus (Linnaeus, 1758)	3D_96_um	ZMB 2520; Atapupu, Timor, Indonesia, Indian Ocean, R/V Gazelle, 1899; 37 mm	7 T PharmaScan; FLASH; TE 6.656 ms, TR 30 ms, NA 12, FOV (3.7 cm)^3, MTX (384 px)^3, TA 14 h 44 min 44 s; (96 µm)^3	Mild susceptibility artifacts caused by paramagnetic substance contained within the digestive tract
Echinocyamidae Lambert & Thiéry, 1914	*Echinocyamus pusillus* (O. F. Müller, 1776)	3D_20_um_Magnevist	ZMB 7410; off Heligoland, North Sea, 2006; 5 mm	17.6 T AVANCE; GEFI; TE 3.27 ms, TR 20 ms, NA 32, FOV 0.5 x 0.45 x 0.45 cm, MTX (256 px)^3, TA 11 h 39 min 3 s; 20 x 18 x 18 µm^3	None
Fibulariidae Gray, 1855	*Fibularia ovulum* Lamarck, 1816	3D_36_um_Magnevist	NMNH E35308; 11°24' N 162°23' E, Parry Island, Eniwetok Atoll, Marshall Islands, Pacific Ocean, 0.5-1 m, July 1959; 8 mm	17.6 T AVANCE; GEFI; TE 2.076 ms, TR 20 ms, NA 60, FOV (0.65 cm)^3, MTX (256)^3, TA 10 h 48 min; (36 µm)^3	Local susceptibility artifacts caused by paramagnetic substance contained within the digestive tract
Laganidae Desor, 1858	*Jacksonaster depressum* (L. Agassiz, 1841)	3D_86_um	BMNH 1932.4.28.227-34; Low Island, Low Isles, Australia, Pacific Ocean, 15 m, Great Barrier Reef Expedition; 32 mm	7 T PharmaScan; FLASH; TE 6.656 ms, TR 30 ms, NA 12, FOV (3.3 cm)^3, MTX (384 px)^3, TA 14 h 44 min 44 s; (86 µm)^3	Mild susceptibility artifacts caused by paramagnetic substance contained within the digestive tract
	Laganum joubini Koehler, 1922	3D_44_um_Magnevist	BMNH 1979.1.25.52-60; Pemba Channel, Tanzania, Indian Ocean; 19 mm	17.6 T AVANCE; GEFI; TE 2.572 ms, TR 20 ms, NA 64, FOV 1.7 x 1.2 x 1.7 cm, MTX 384 x 270 x 384 px, TA 36 h	Mild susceptibility artifacts caused by paramagnetic substance contained within the digestive

				51 min 50 s; (44 µm)^3	tract
	Laganum laganum (Leske, 1778)	3D_81_um	NMNH E09175; Romblon, Philippines, Pacific Ocean, R/V Albatross, March 1908; 28 mm	7 T PharmaScan; FLASH; TE 6.656 ms, TR 30 ms, NA 12, FOV (3.12 cm)^3, MTX (384 px)^3, TA 14 h 44 min 44 s; (81 µm)^3	Mild susceptibility artifacts caused by paramagnetic substance contained within the digestive tract
	Peronella lesueuri (L. Agassiz, 1841)	3D_81_um	MNHN EcEh79; Singapore, Pacific Ocean; 30 mm	7 T PharmaScan; FLASH; TE 6.656 ms, TR 30 ms, NA 12, FOV (3.12 cm)^3, MTX (384 px)^3, TA 14 h 44 min 44 s; (81 µm)^3	None
		3D_81_um_Magnevist	MNHN EcEh79; Singapore, Pacific Ocean; 30 mm	7 T PharmaScan; FLASH; TE 6.656 ms, TR 30 ms, NA 12, FOV (3.12 cm)^3, MTX (384 px)^3, TA 14 h 44 min 44 s; (81 µm)^3	Mild susceptibility artifacts caused by paramagnetic substance contained within the digestive tract
	Peronella orbicularis (Leske, 1778)	3D_81_um	MNHN EcEh77; New Caledonia, Pacific Ocean, 1928; 22 mm	7 T PharmaScan; FLASH; TE 6.656 ms, TR 30 ms, NA 12, FOV (3.12 cm)^3, MTX (384 px)^3, TA 14 h 44 min 44 s; (81 µm)^3	None
		3D_81_um_Magnevist	MNHN EcEh77; New Caledonia, Pacific Ocean, 1928; 22 mm	7 T PharmaScan; FLASH; TE 6.656 ms, TR 30 ms, NA 12, FOV (3.12 cm)^3, MTX (384 px)^3, TA 14 h 44 min 44 s; (81 µm)^3	None
Rotulidae Gray, 1855	*Rotula deciesdigitatus* (Leske, 1778)	3D_81_um	ZMB 2169; Monrovia, Liberia, 11-18 m, R/V Gazelle, February 1876; 24 mm	7 T PharmaScan; FLASH; TE 6.656 ms, TR 30 ms, NA 12, FOV (3.12 cm)^3, MTX (384 px)^3, TA 14 h 44 min 44 s; (81 µm)^3	Strong susceptibility artifacts caused by air bubbles and paramagnetic substance contained within the digestive tract
Echinarachniidae Lambert in Lambert & Thiéry, 1914	*Echinarachnius parma* (Lamarck, 1816)	3D_44_um_Magnevist	BMNH 55.10.3.125; Ecuador, Pacific Ocean; 21 mm	17.6 T AVANCE; GEFI; TE 2.572 ms, TR 20 ms, NA 26, FOV 1.7 x 1.2 x 1.7 cm, MTX 384 x 272 x 384 px, TA 15 h 5 min 12 s; (44 µm)^3	Strong susceptibility artifacts caused by air bubbles and paramagnetic substance contained within the digestive tract
		3D_81_um	ZSM 20011676; East Asia, Pacific Ocean, 1900; 24 mm	7 T PharmaScan; FLASH; TE 6.656 ms, TR 30 ms, NA 12, FOV (3.12 cm)^3, MTX (384 px)^3, TA 14 h 44 min 44 s; (81 µm)^3	Local susceptibility artifacts caused by paramagnetic substance contained within the digestive tract
		3D_81_um_Magnevist	ZSM 20011676; East Asia, Pacific Ocean, 1900; 24 mm	7 T PharmaScan; FLASH; TE 6.656 ms, TR 30 ms, NA 12, FOV (3.12 cm)^3, MTX (384 px)^3, TA 14 h 44 min	Local susceptibility artifacts caused by paramagnetic substance contained within the digestive

				44 s; (81 µm)^3	tract
Astriclypeidae Stefanini, 1912	*Astriclypeus manni* Verrill, 1867	3D_47_um_Magnevist	MMBS; Misaki, Japan, Pacific Ocean, 20 m, March 2013; 16 mm	9.4 T BioSpec; RARE; TE 11.22 ms, TR 1500 ms, NA 8, FOV 2.4 x 2.4 x 0.6 cm, MTX 512 x 512 x 128 px, TA 15 h 21 min 36 s; (47 µm)^3	None
	Echinodiscus auritus Leske, 1778	3D_47_um_Magnevist	ZMK Mortensen collection; Maroon Point, Mauritius, Indian Ocean, 37-46 m, October 1929; 16 mm	9.4 T BioSpec; RARE; TE 11.22 ms, TR 1500 ms, NA 8, FOV 2.4 x 2.4 x 0.6 cm, MTX 512 x 512 x 128 px, TA 15 h 21 min 36 s; (47 µm)^3	Mild susceptibility artifacts caused by paramagnetic substance contained within the digestive tract
	Echinodiscus bisperforatus Leske, 1778	3D_47_um_Magnevist	ZMK Mortensen collection; Polana Beach, Mozambique, Indian Ocean, September 1929; 15 mm	9.4 T BioSpec; RARE; TE 11.22 ms, TR 1500 ms, NA 8, FOV 2.4 x 2.4 x 0.6 cm, MTX 512 x 512 x 128 px, TA 15 h 21 min 36 s; (47 µm)^3	Strong susceptibility artifacts caused by paramagnetic substance contained within the digestive tract
Urechinidae Duncan, 1889	*Antrechinus mortenseni* (David & Mooi, 1990)	3D_81_um	ZMH E7381; 61°23'40'' S 55°26'99'' W, off Elephant Island, Southern Ocean, 286 m, R/V Polarstern, LAMPOS PS 61 Expedition Station 61/253-1; 29 mm	7 T PharmaScan; FLASH; TE 6.656 ms, TR 30 ms, NA 12, FOV (3.12 cm)^3, MTX (384 px)^3, TA 14 h 44 min 44 s; (81 µm)^3	Strong susceptibility artifacts caused by air bubbles and paramagnetic substance contained within the digestive tract
	Urechinus naresianus A. Agassiz, 1879	3D_81_um	ZSM 20012380; 41°24'45'' N 65°35'30'' W, S off New Scotia, Atlantic Ocean, 2272 m; 27 mm	7 T PharmaScan; FLASH; TE 6.656 ms, TR 30 ms, NA 12, FOV (3.12 cm)^3, MTX (384 px)^3, TA 14 h 44 min 44 s; (81 µm)^3	Strong susceptibility artifacts caused by paramagnetic substance contained within the digestive tract
		3D_81_um_Magnevist	ZSM 20012380; 41°24'45'' N 65°35'30'' W, S off New Scotia, Atlantic Ocean, 2272 m; 27 mm	7 T PharmaScan; FLASH; TE 6.656 ms, TR 30 ms, NA 12, FOV (3.12 cm)^3, MTX (384 px)^3, TA 14 h 44 min 44 s; (81 µm)^3	Strong susceptibility artifacts caused by paramagnetic substance contained within the digestive tract
Plexechinidae Mooi & David, 1996	*Plexechinus planus* (Mironov, 1978)	3D_81_um	ZMH E7345; 73°24' S 22° 35' W, Weddell Sea, Southern Ocean, 1353 m, R/V Polarstern, ANT XV/3 Expedition Station 48-088; 30 mm	7 T PharmaScan; FLASH; TE 6.656 ms, TR 30 ms, NA 12, FOV (3.12 cm)^3, MTX (384 px)^3, TA 14 h 44 min 44 s; (81 µm)^3	Strong susceptibility artifacts caused by paramagnetic substance contained within the digestive tract
		3D_81_um_Magnevist	ZMH E7345; 73°24' S 22° 35' W, Weddell Sea, Southern Ocean, 1353 m, R/V	7 T PharmaScan; FLASH; TE 6.656 ms, TR 30 ms, NA 12, FOV (3.12 cm)^3, MTX (384 px)^3, TA 14 h 44 min	Strong susceptibility artifacts caused by paramagnetic substance contained within the digestive

			Polarstern, ANT XV/3 Expedition Station 48-088; 30 mm	44 s; (81 µm)^3	tract
Pourtalesiidae A. Agassiz, 1881	*Pourtalesia jeffreysi* Thomson, 1873	3D_81_um	ZSM 20011456; Norwegische Nordmeer-Expedition; 28 mm	7 T PharmaScan; FLASH; TE 6.656 ms, TR 30 ms, NA 12, FOV (3.12 cm)^3, MTX (384 px)^3, TA 14 h 44 min 44 s; (81 µm)^3	Strong susceptibility artifacts caused by paramagnetic substance contained within the digestive tract
		3D_81_um_Magnevist	ZSM 20011456; Norwegische Nordmeer-Expedition; 28 mm	7 T PharmaScan; FLASH; TE 6.656 ms, TR 30 ms, NA 12, FOV (3.12 cm)^3, MTX (384 px)^3, TA 14 h 44 min 44 s; (81 µm)^3	Strong susceptibility artifacts caused by paramagnetic substance contained within the digestive tract
	Pourtalesia wandeli Mortensen, 1905	3D_86_um	BMNH 1976.7.30.76-95; 60°07' N 19°27' W, Iceland Basin, Atlantic Ocean, 2636-2646 m, Discovery Investigations Expedition Station 7709, 1976; 33 mm	7 T PharmaScan; FLASH; TE 6.656 ms, TR 30 ms, NA 12, FOV (3.3 cm)^3, MTX (384 px)^3, TA 14 h 44 min 44 s; (86 µm)^3	Strong susceptibility artifacts caused by paramagnetic substance contained within the digestive tract
		3D_86_um_Magnevist	BMNH 1976.7.30.76-95; 60°07' N 19°27' W, Iceland Basin, Atlantic Ocean, 2636-2646 m, Discovery Investigations Expedition Station 7709, 1976; 33 mm	7 T PharmaScan; FLASH; TE 6.656 ms, TR 30 ms, NA 12, FOV (3.3 cm)^3, MTX (384 px)^3, TA 14 h 44 min 44 s; (86 µm)^3	Strong susceptibility artifacts caused by paramagnetic substance contained within the digestive tract
Schizasteridae Lambert, 1905	*Abatus cavernosus* (Philippi, 1845)	3D_81_um	ZMB 5854; Bassin de la Gazelle, Kerguelen Islands, Southern Ocean, Deutsche Tiefsee-Expedition Station 160, 1898; 28 mm	7 T PharmaScan; FLASH; TE 6.656 ms, TR 30 ms, NA 12, FOV (3.12 cm)^3, MTX (384 px)^3, TA 14 h 44 min 44 s; (81 µm)^3	Mild susceptibility artifacts caused by paramagnetic substance contained within the digestive tract
		3D_81_um_Magnevist	ZMB 5854; Bassin de la Gazelle, Kerguelen Islands, Southern Ocean, Deutsche Tiefsee-Expedition Station 160, 1898; 28 mm	7 T PharmaScan; FLASH; TE 6.656 ms, TR 30 ms, NA 12, FOV (3.12 cm)^3, MTX (384 px)^3, TA 14 h 44 min 44 s; (81 µm)^3	Mild susceptibility artifacts caused by paramagnetic substance contained within the digestive tract
Spatangidae Gray, 1825	*Spatangus purpureus* O. F. Müller, 1776	3D_81_um	ZMB 3236; 54°55' N 06°40' E, North Sea, 35-40 m, Sophie Nordsee-Expedition Station	7 T PharmaScan; FLASH; TE 6.656 ms, TR 30 ms, NA 12, FOV (3.12 cm)^3, MTX (384 px)^3, TA 14 h 44 min	Strong susceptibility artifacts caused by paramagnetic substance contained within the digestive

			124; 28 mm	44 s; (81 µm)^3	tract
		3D_81_um_Magnevist	ZMB 3236; 54°55' N 06°40' E, North Sea, 35-40 m, Sophie Nordsee-Expedition Station 124; 28 mm	7 T PharmaScan; FLASH; TE 6.656 ms, TR 30 ms, NA 12, FOV (3.12 cm)^3, MTX (384 px)^3, TA 14 h 44 min 44 s; (81 µm)^3	Strong susceptibility artifacts caused by paramagnetic substance contained within the digestive tract
Maretiidae Lambert, 1905	*Nacospatangus alta* (A. Agassiz, 1863)	3D_81_um	ZSM 20011608; Sri Lanka, Indian Ocean, 1887; 30 mm	7 T PharmaScan; FLASH; TE 6.656 ms, TR 30 ms, NA 12, FOV (3.12 cm)^3, MTX (384 px)^3, TA 14 h 44 min 44 s; (81 µm)^3	Strong susceptibility artifacts caused by paramagnetic substance contained within the digestive tract
		3D_81_um_Magnevist	ZSM 20011608; Sri Lanka, Indian Ocean, 1887; 30 mm	7 T PharmaScan; FLASH; TE 6.656 ms, TR 30 ms, NA 12, FOV (3.12 cm)^3, MTX (384 px)^3, TA 14 h 44 min 44 s; (81 µm)^3	Strong susceptibility artifacts caused by paramagnetic substance contained within the digestive tract
Loveniidae Lambert, 1905	*Echinocardium cordatum* (Pennant, 1777)	3D_81_um	ZMB 7407; off Heligoland, North Sea, 2006; 29 mm	7 T PharmaScan; FLASH; TE 6.656 ms, TR 30 ms, NA 12, FOV (3.12 cm)^3, MTX (384 px)^3, TA 14 h 44 min 44 s; (81 µm)^3	Strong susceptibility artifacts caused by paramagnetic substance contained within the digestive tract
		3D_117_um_Magnevist	ZMB 7407; off Heligoland, North Sea, 2006; 29 mm	7 T PharmaScan; FLASH; TE 6 ms, TR 30 ms, NA 8, FOV (3 cm)^3, MTX (256 px)^3, TA 4 h 22 min 8 s; (117 µm)^3	Strong susceptibility artifacts caused by paramagnetic substance contained within the digestive tract

2D = two-dimensional, 3D = three-dimensional, BMNH = British Museum of Natural History (London, UK), CASIZ = California Academy of Sciences Invertebrate Zoology (San Francisco, CA, USA), FLASH = fast low angle shot, FOV = field of view, GEFI = gradient echo fast imaging, MMBS = Misaki Marine Biological Station (Misaki, Japan), MNHN = Muséum national d'Histoire naturelle (Paris, France), MTX = matrix size, NA = number of averages, NHMW = Naturhistorisches Museum Wien (Vienna, Austria), NIWA = National Institute of Water and Atmospheric Research (Auckland, New Zealand), NMNH = National Museum of Natural History (Washington, DC, USA), NS = slice number, PPSIO = P. P. Shirshov Institute of Oceanology (Moscow, CIS), RARE = rapid acquisition with relaxation enhancement, R/V = research vessel, ST = slice thickness, TA = acquisition time, TE = echo time, TR = repetition time, ZMB = Systematische Zoologie am Museum für Naturkunde (Berlin, Germany), ZMH = Zoologisches Institut und Museum Hamburg (Hamburg, Germany), ZMK = Zoologisk Museum København (Copenhagen, Denmark), ZSM = Zoologische Staatssammlung München (Munich, Germany).

Specimen preparation

Museum specimens were mostly fixed in formalin and then conserved in alcohol, although in some cases the exact mode of fixation could not be determined. For MRI, all specimens had to be lowered down to distilled water in a gradual ethanol series. The specimens where placed either inside a custom-built plexiglas chamber, inside a 50 ml Falcon tube, or inside nuclear magnetic resonance tubes with diameters between 5 and 20 mm, depending on the size of the specimen. Small specimens were placed in low-melting agarose to prevent movement artifacts, while some of the larger specimens were immobilized using glass or plastic rods. On some specimens, spines had to be dressed to ensure a tight fit inside the tubes. Prior to scanning, samples were degassed at 50 mbar. Most museum specimens have been stored in separate jars for potential later re-scanning.

Specimen contrasting

Magnevist (Bayer HealthCare, Leverkusen, Germany), a gadolinium-based non-selective contrast agent, was added at a final concentration of 2 mM. This concentration had been tested successfully in preliminary studies. The addition of the contrast agent to the museum specimens was discussed beforehand with the curators who judged the risk to the specimens' integrity as negligible.

Specimen scanning

The scans deposited online and listed in Supplementary Table 1 were acquired at around 18°C. Data acquisition and image processing were carried out using the software Paravision 3.0 and 4.0 (Bruker Biospin GmbH). In several cases, image processing involved zero filling to a larger matrix prior to Fourier transformation. Scanning protocols employed different 2D and 3D imaging sequences, as detailed in Supplementary Table 1. Scanning parameters depended on the properties of the scanner as well as specimen size. Scanning parameters are listed in Supplementary Table 1, but can also be found in the metadata files deposited online.

Data availability

Every scan resulted in a set of raw and metadata files that within the respective scan folder (e.g. Strongylocentrotus_purpuratus_3D_81_um) can be found inside the subfolder labeled 'RAW'. In parallel to the raw image data, an image stack in TIFF format at 8-bit dynamic range is provided in the subfolder labeled 'TIFF'. All scan folders were packed using tape archiver (Tar, .tar, [2]) and compressed using GNU zip (Gzip, .gz, [3]). For convenient data download, all scan folders were packed into a single, 39 GB large tape archive, which is also available online. The size of the entire uncompressed dataset amounts to 92 GB. Furthermore, in addition to the scan folders and based on each TIFF stack, animated graphics interchange format (GIF, .gif) files were generated and deposited online. These GIF files permit rapid access to the image data contained within each scan. A subset of 43 TIFF 3D image stacks was previously uploaded to the online repository MorphDBase [4]. However, due to size limitations, this dataset was not accompanied by the essential raw data [5].

Data requirements

A computer system with about 1 GB of random access memory (RAM) and 1 GB of video random access memory (VRAM) should be used for smooth data handling. Following download, the metadata files (e.g., 'method' or 'reco'), raw image data files (i.e., '2dseq'), and derived image data files (i.e., 'TIFF') can all be accessed using the platform-independent, Java-based imaging software ImageJ (additionally requires installation of the BrukerOpener plugin, [6]). In addition, we recommend using the ImageJ Volume Viewer plugin [7] for interactive rotation and slicing of the scan data. However, numerous other 2D and 3D visualization tools can be obtained online [8].

To open the metadata files contained within the 'RAW' folder, simply drag-and-drop the respective file onto the main menu panel in ImageJ (requires installation of the BrukerOpener plugin, see above). The same procedure can be used to open the raw image data contained within the '2dseq' file or the free induction decay data available in the 'fid' file. Note that the BrukerOpener plugin uses the lookup table (LUT) 'Fire' to display raw image data - follow the 'Image:Lookup Tables:Grays' command chain to obtain the original grayscale data display. In order to open

one of the TIFF image stacks, choose the 'File:Import:Image Sequence' command chain and then select the first TIFF file from the folder. Note that all raw image data gathered on the 7 T PharmaScan need to be rescaled to 384 x 384 pixel dimensions following opening in ImageJ - use the 'Image:Adjust:Size' command chain to alter the respective image stack.

Table S2. Overview of Bruker MRI file types.

The following table lists the names and descriptions for most of the files typically created during an MRI scan using Bruker systems.

**Filename**	**Description**
2dseq	Processed image data expressed in a raw binary format without header
acqp	Base-level acquisition parameters
AdjStatePerScan	Last adjustments made to the scan
AdjStatePerStudy	Last adjustments made to the study
d3proc	Description of the image data contained in the 2dseq file
fid	The raw, unreconstructed free induction decay data
id	Unique dataset identification
meta	Used for backward compatibility between different Bruker software
method	High-level acquisition parameters
procs	Used for backward compatibility between different Bruker software
pulseprogram	Source code for the pulse sequence used in the acquisition
reco	Input and output parameters for the reconstruction process
roi	Summary of region of interest definitions for the scan
spnam	Shape pulse definition used during acquisition
subject	Subject and study parameters
visu_pars	Parameters for postprocessing, conversion, and data display

References

1. Ziegler A, Kunth M, Mueller S, Bock C, Pohmann R, Schröder L, Faber C, Giribet G: **Application of magnetic resonance imaging in zoology.** *Zoomorphology* 2011, **130:**227-254. doi:10.1007/s00435-011-0138-8

2. Free Software Foundation: **GNU Tar.** [http://www.gnu.org/software/tar/]

3. Free Software Foundation: **GNU Gzip.** [http://www.gnu.org/software/gzip/]

4. Vogt L, Grobe P: **MorphDBase.** [https://www.morphdbase.de/]

5. Ziegler A: **Non-invasive imaging and 3D visualization techniques for the study of sea urchin internal anatomy.** *PhD thesis*. Freie Universität Berlin, Fachbereich Biologie, Chemie und Pharmazie; 2008. [http://www.diss.fu-berlin.de/diss/receive/FUDISS_thesis_000000006510]

6. Manz B: **BrukerOpener.** [http://rsbweb.nih.gov/ij/plugins/bruker.html]

7. Barthel KU: **Volume Viewer 2.01.** [http://rsb.info.nih.gov/ij/plugins/volume-viewer.html]

8. Elicieri KW, Berthold MR, Goldberg IG, Ibánez L, Manjunath BS, Martone ME, Murphy RF, Peng H, Plant AL, Roysam BR, Stuurman N, Swedlow JR, Tamancak P, Carpenter AE: **Biological imaging software tools.** *Nature Meth* 2012, **9**:697-710. doi:10.1038/nmeth.2084
